# Investigating Nutrient Supply Effects on Plant Growth and Seed Nutrient Content in Common Bean

**DOI:** 10.3390/plants11060737

**Published:** 2022-03-10

**Authors:** Millicent R. Smith, Barbara Elias Reis Hodecker, David Fuentes, Andrew Merchant

**Affiliations:** School of Life and Environmental Sciences, Faculty of Science, The University of Sydney, Sydney, NSW 2006, Australia; millicent.smith@uq.edu.au (M.R.S.); beliasreis@gmail.com (B.E.R.H.); david.fuentes@sydney.edu.au (D.F.)

**Keywords:** amino acid, legume, nutrition, *Phaseolus vulgaris*, phloem

## Abstract

Low soil fertility commonly limits growth and yield production of common bean (*Phaseolus vulgaris* L.) in tropical regions. Impacts of nutrient limitations on production volume are well studied and are a major factor in reducing crop yields. This study characterised the impact of reduced nutrient supply on carbon assimilation and nutrient content of leaf, phloem sap and reproductive tissues of common bean grown in a controlled environment in order to detect chemical markers for changes in nutritional content. Leaf gas exchange measurements were undertaken over plant development to characterise changes to carbon assimilation under reduced nutrient supply. Samples of leaf, phloem sap and pod tissue of common bean were analysed for carbon isotope discrimination, mineral nutrient content, and amino acid concentration. Despite declines in nutrient availability leading to decreased carbon assimilation and reductions in yield, amino acid concentration was maintained in the pod tissue. Common bean can maintain the nutritional content of individual pods under varying nutrient availabilities demonstrating the resilience of processes determining the viability of reproductive tissues.

## 1. Introduction

Common bean (*Phaseolus vulgaris* L.), is the most important grain legume for human consumption [1] and a staple food for millions of people, primarily in tropical Latin America, South and East Africa [2]. Low soil fertility is a major constraint to yields in these regions [3] as production is often restricted to marginal land [4]. High costs of synthetic fertilisers render them largely unavailable to many common bean production systems compared to high-value crops such as maize [3,5]. Although legumes are able to fix atmospheric nitrogen, symbiotic nitrogen fixation rarely satisfies the full nitrogen demand of common bean [6].

The influence of low soil fertility on plant growth in common bean is well understood [7,8,9] and unlike other stresses, such as drought or disease, low soil fertility is an ongoing issue in tropical regions, irrespective of season [6,10]. Low soil fertility impacts on all aspects of plant growth including carbon assimilation [11], root formation [12], symbiotic nitrogen fixation [13,14] and yield [8]. Nevertheless, the impact of low soil fertility on nutrient content, particularly that of the seed, is less understood. This has important implications for the hundreds of millions of consumers who rely on common bean as a vital source of nutrients [1].

Our capacity to predict the quality of seed that will be produced from a given crop is limited by our understanding of yield development and processes determining nutrient accumulation in reproductive tissues. Nutritional content for common bean has been described previously [1] with vast repositories of nutrient information freely available through large databases see for example, Reference [15]. However, research has tended to focus on protein, iron and zinc availability due to their relative importance in the recognised deficiencies of modern human nutrition. Genotypic variability of nutrient quality for protein, iron and zinc has been highlighted previously and targeted in breeding efforts to increase bioavailability [16,17,18]. To target declining nutrient content prior to harvest, diagnostic tools based on analysis of vegetative tissues would be useful to inform management prescriptions to mitigate the effects of low nutrient supply during key stages of reproductive development. Measuring the soluble, and thus transported, pool within vegetative tissues may allow for the characterisation of carbon, water and nutrient status of the plant and would serve as a beneficial tool to assist with decision making in productions systems.

Few studies have investigated nutrient concentrations in vegetative and reproductive plant pools concurrently [19] outside early findings by Hocking and Pate [20] who detected changes in mineral nutrient concentration in the leaf and phloem tissue of legume species during pod development. Here we investigate the impact of reduced nutrient supply on common bean over the course of reproductive development in controlled conditions. Specifically, we address the following hypothesis: (1) reduced nutrient supply will reduce plant gas exchange and that the magnitude of this reduction will increase over the course of development; (2) reduced nutrient supply will decrease yield volume; (3) reduced nutrient supply will alter the composition and decrease the concentration of metabolites (amino acids, mineral nutrients) in the leaf, phloem and pod soluble fractions and these will be consistent throughout development; (4) reduced nutrient supply will alter the composition, and decrease the concentration of metabolites (amino acids, mineral nutrients) in pod tissues and (5) and changes in total amino acid and mineral nutrients from digested pod samples will correspond to changes in soluble extracts of pod tissues.

## 2. Results

### 2.1. Effect of Nutrient Deficiency on Carbon Assimilation and Yield

Leaf level gas exchange displayed no difference between treatments across development, except during the pod maturity stage, where a significant reduction (63%) in photosynthetic rate (A) for the low nutrient supply treatment, compared to control (*p* < 0.05), was observed (Table 1). Stomatal conductance at this stage reduced by 25% and 50 % from the control and medium nutrient supply treatments, respectively, to the low treatment (*p* < 0.05). Sub-stomatal concentration of CO_2_ (c_i_) did not vary with development or treatment (Table 1).

**Figure 1:**
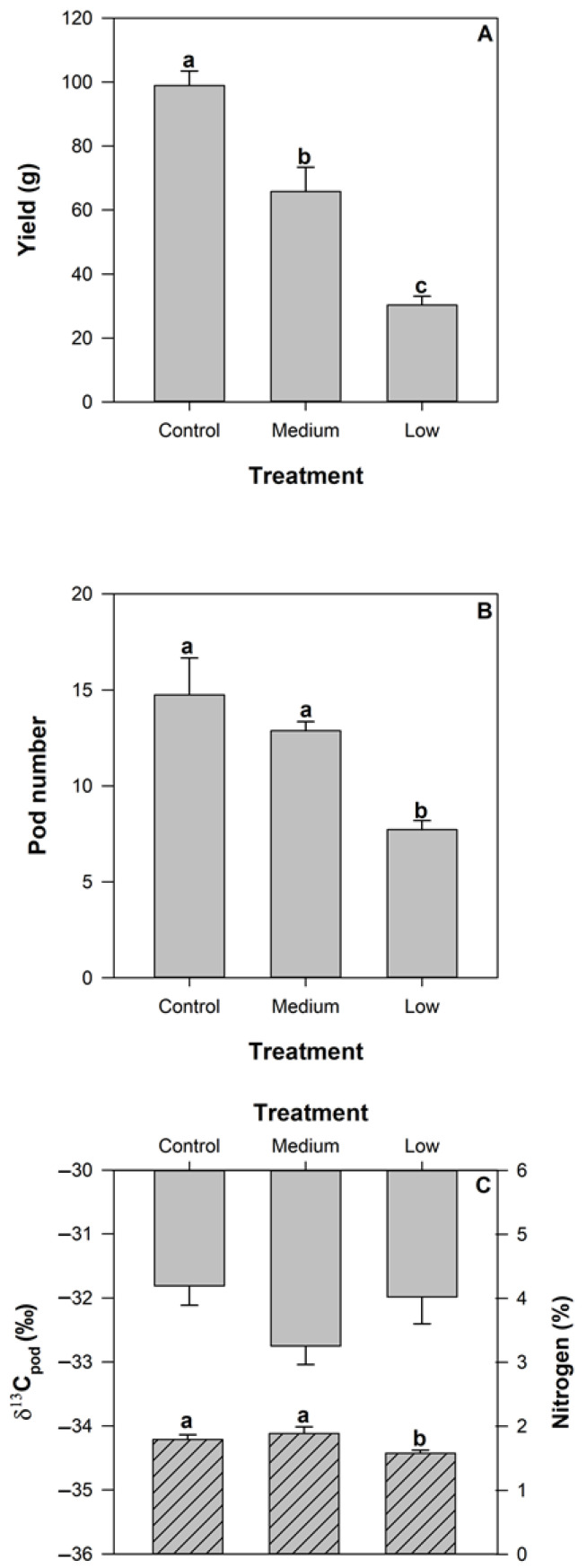
Yield (g/plant) (**A**), pod number/plant (**B**), carbon isotope abundance (grey columns, left axis) and nitrogen (hashed columns, right axis) (**C**) of pod material collected at pod maturity for common bean subject to control (100%), medium (50%) and low (10%) nutrient supply. Standard error bars are shown where *n* = 8. Statistically significant differences between treatments were detected for yield, pod number and nitrogen content (denoted by multiple comparisons lettering).

Yield (dry weight of pod and seed) at pod maturity significantly differed with all treatments (*p* < 0.05) with a reduction of approximately 75% and 50% between the control and medium treatments, respectively, compared to plants that received low nutrient supply (Figure 1A). Pod number per plant also differed significantly (approx. 40%) between the control compared to the low treatment (Figure 1B) and was similar to the medium nutrient supply treatment.

### 2.2. Impact of Nutrient Deficiency on Carbon Isotope Abundance and Carbon and Nitrogen Content of Plant Tissues

Carbon isotope abundance did not significantly differ between treatments in the leaf material (Table 1) or the pod material (Figure 1). Mature pods exhibited a statistically significant difference in nitrogen content between the control and medium treatment, approximately 18% higher, compared to the low nutrient supply treatment (Figure 1C).

### 2.3. Impact of Nutrient Deficiency on Nutrient Concentrations and Content in Plant Tissues

**Figure 2:**
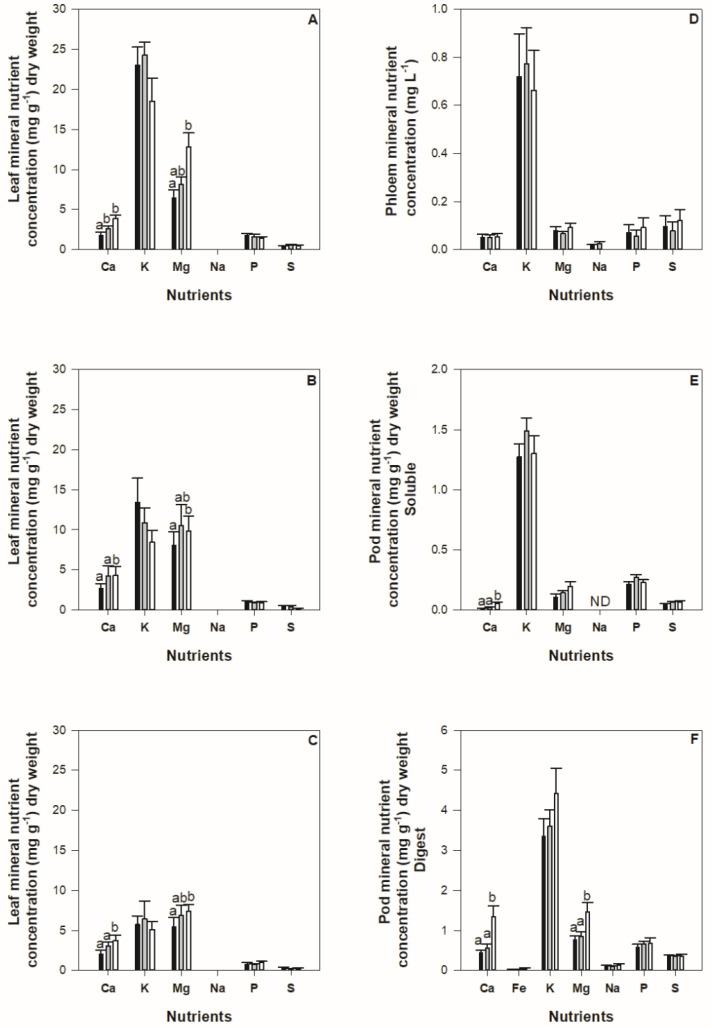
Concentration of soluble nutrients in leaf (mg g^−1^) for flowering (**A**), mid-pod fill (**B**) and pod maturity (**C**) stage and phloem (mg L^−1^) (**D**), soluble pod (mg g^−1^) (**E**), and digest pod (**F**) for pod maturity stage. Nutrients; calcium, iron, potassium, magnesium, phosphorus, sulphur and zinc found in common bean subject to control (100%) nutrient availability (black columns), medium (50%) nutrient availability (grey columns) and low (10%) nutrient availability (white columns). Standard error bars are shown where *n* = 8. Statistically significant differences are denoted with multiple comparisons lettering. Iron and zinc were not detected in the soluble leaf, phloem or pod pool.

In the leaf tissue, concentration of nutrients decreases across development with statistically significant differences in potassium (K), magnesium (Mg) and phosphorus (P) found across development (*p* < 0.05) (Figure 2A–C). Reduced nutrient supply influenced leaf calcium (Ca) and Mg concentration within each development stage (*p* < 0.05), where higher concentrations were found in plants under low nutrient availability. No statistical differences were observed for K, P and sulphur (S) leaf concentration between treatments at the same development stage.

The concentration of nutrients is higher in leaves compared to pods (Figure 2). Overall K was present in the highest concentration in all treatments and development periods, while Mg is also prominent in the soluble leaf tissue. Despite differences in concentration of nutrients among treatments in the pod material, a statistically significant difference was only found between the control and medium nutrient supply treatment compared to the low nutrient supply treatment for Ca and Mg (Figure 2E,F). In the phloem, no significant statistical differences on nutrient concentration were observed between treatments for any nutrients (Figure 2D).

### 2.4. Impact of Nutrient Deficiency on Amino Acid Concentration

**Figure 3:**
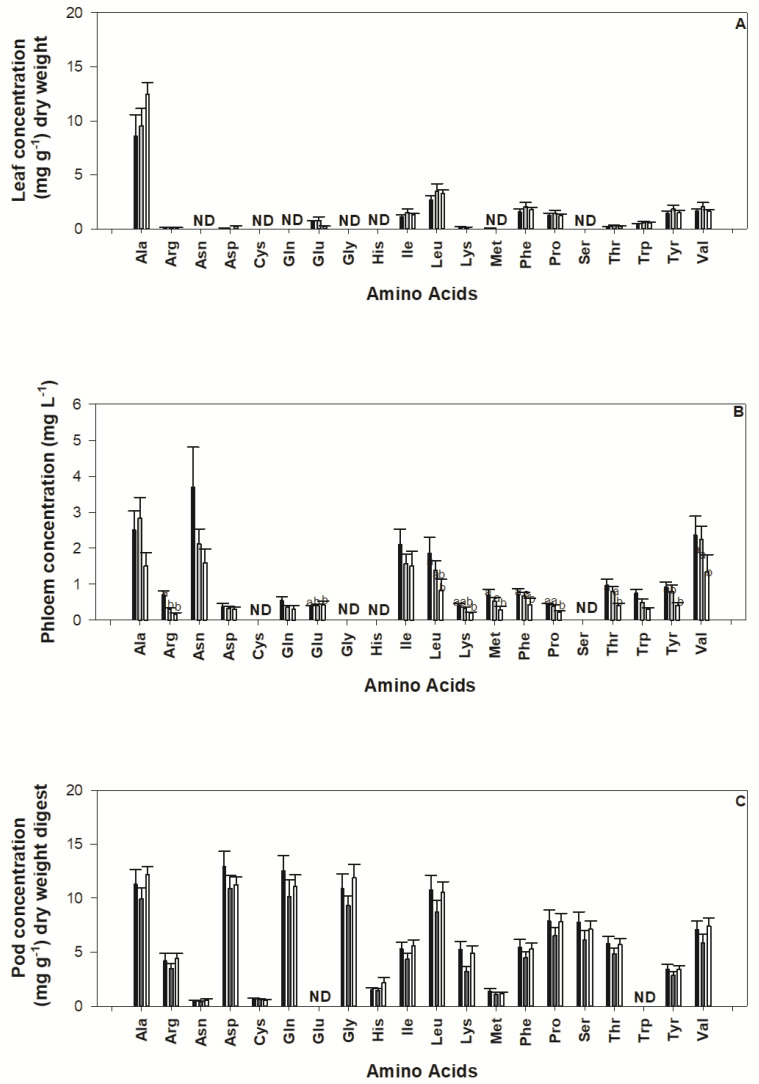
Concentration of amino acids; alanine, arginine, asparagine, aspartic acid, cysteine, glutamine, glutamic acid, glycine, histidine, isoleucine, leucine, lysine, methionine, phenylalanine, proline, serine, threonine, tryptophan, tyrosine and valine found in common bean soluble leaf extract (**A**), phloem (**B**) and total digested pods (**C**) subject to control (100%) nutrient availability (black columns), medium (50%) nutrient availability (grey columns) and low (10%) nutrient availability (white columns) for pod maturity stage. Standard error bars are shown where *n* = 8. Statistically significant differences in treatment for the phloem pool were found in treatment and are denoted with multiple comparisons lettering.

Amino acid concentration and composition varies between organs, development stages and with treatment (Figure 3). Alanine was detected in high concentration among all organs and treatments. In the soluble leaf tissue extract, no statistically significant difference between treatments for any amino acids were detected (*p* > 0.05) (Figure 3A). In the phloem, statistically significant differences were observed for amino acids detected between the control treatment and reduced nutrient supply treatments (as denoted by the multiple comparisons shown on Figure 3B). In the digested pod tissue, no statistically significant treatment differences were found (Figure 3C).

## 3. Discussion

### 3.1. Nutrient Supply Impacted Plant Growth and Yield Production

Reduced nutrient supply significantly decreased yield in agreement with previous observations across a range of both controlled environment and field conditions [21,22]. Reduced nutrient supply also influenced leaf level gas exchange at pod maturity (Table 1). These observations, combined with the lack of response in c_i_ (Table 1), suggest that aspects of leaf biochemistry, such as leaf nitrogen concentration (Table 1) and physiology, acted together to maintain homeostatic control in response to both treatment and developmental effects. We also observed moderate declines in specific leaf area (data not shown), and statistically significant reductions in yield and pod number (Figure 1A,B) with treatment. Whilst an influence of reduced nutrient supply was observed, consistent patterns in amino acids and mineral nutrients between plant tissues were not detected. Plants appeared to be able to buffer the concentration of amino acids and mineral nutrients among tissues under the magnitude of nutrient limitations achieved in this study.

### 3.2. Nutrient Supply Did Not Alter Yield Nutrient Concentration

Reduced nutrient supply did not significantly impact the concentration of amino acids in the digested pod tissue (Figure 3) or key micronutrient iron (Figure 2F) even though reductions in pod nutrient content were observed under low nutrient supply. Decreases in Ca and Mg concentration in control plant leaf tissue may be due to dilution caused by higher plant growth under normal nutrient supply.

Given the importance of common bean in consumers’ diets and lack of inputs available in some production systems [1]; the resilience of protein and iron concentration in the reproductive tissue to changes in nutrient availability is encouraging. The influence of reduced nutrient supply on the concentration of other nutrients such as Ca and Mg were observed in the pod tissue at maturity (Figure 2E,F). Reduced nutrient supply also promoted a significant reduction in the number of pods produced, suggesting that the plant altered pod development to maintain protein concentration in the reproductive tissue.

Substantial decreases in nutrient concentrations were detected in the leaf tissue as reproductive tissue developed (Figure 2A–C) similar to those uncovered by Hocking and Pate [20] in *Lupinus* spp., *Pisum sativum*, and by Garcia and Grusak [19] in *Medicago truncatula*. The decrease in leaf nutrient concentration for mobile nutrients such as P, K and Mg, observed in this study, throughout development is likely due to remobilisation of leaf resources for allocation to developing reproductive tissues, perhaps in this case exacerbated by the treatment effects. Remobilisation of leaf nutrient reserves is well known [23,24], however the magnitude of this process, and its resilience to the effects of reduced nutrient supply are poorly characterised. In the present study, characterising whole plant allocation patterns were restricted by our pot-based approach and its known limitations see for example, Reference [25]. Further exploration of this process under field conditions, and the magnitude to which it confers resilience to the development of reproductive tissues is required.

Decreasing nutrient supply despite reductions to yield and pod number did not influence protein and some macronutrient concentrations within the reproductive tissue. This suggests that the biological imperative to reproduce, requiring key nutrients for seed germination to occur, may strongly influence the resilience of nutritional quality. The resilience of nutrient and amino acid concentration in reproductive tissue in this study contrasts with other studies which have described changes in nutrient availability and uptake in response to drought [26] and nutritional quality when grown under elevated [CO_2_] [21]. Whilst further investigations under more realistic growth conditions are required, a more fundamental understanding of the process of nutrient transfer from the leaf to the pod would be beneficial. Recent research by Tegeder and Hammes [27] into amino acid transport along with micronutrient transport [28] is developing an improved understanding of the processes involved in loading different nutrient pools into the seed. Limited understanding of transport mechanisms from vegetative tissue to the pod wall and on to the developing seed, as described by Garcia and Grusak (2015), alongside ontological changes; has substantially and negatively impacted our ability to make improvements to seed nutrient concentration [19,29,30]. The current model of nutrient accumulation from leaves to the pod wall then on to the developing seed as described by Garcia and Grusak [19] was unable to be investigated in this study as the entire reproductive tissue (pod wall and included seeds) were analysed as a whole. However, the resilience of the reproductive tissue to reduced nutrient supply in this study supports the notion that the pod wall is crucial in supplying nutrients to the seed. Further exploration of approaches to enhance export from pod walls and leaves would likely increase nutrient content in the seed. It is known that both leaf tissue and pod walls are important storage areas for mineral nutrients and amino acids and exploration of approaches to enhance export to developing seeds in legume species would likely be valuable see, References [19,29] although dependent on source-sink dynamics [31].

### 3.3. Soluble Extracts Did Not Reflect Digested Samples and Were Not Consistent throughout Development or Tissue Type

Results obtained from soluble extracts were not similar to those from digested samples (see, Figure 2). This is somewhat expected as photoassimilates are transported throughout the plant in the form of reduced compounds as a means of ‘packaging’ for long distance movement [32] and the digestion of pod material broke down protein polymers into constituent amino acids [33]. While direct comparison between plant metabolite pools was restricted in this study due to the difficulties in sample collection on such small plant organs, phloem nutrients have been analysed on a concentration of volume (mg L^−1^) rather than weight basis (mg g^−1^), patterns between the phloem pool and the soluble leaf extract are similar (Figure 2 and Figure 3). Nevertheless, the array of amino acids detected in the phloem pool reflects extraction of living tissue (companion cells and periderm) rather than solely the transported phloem pool which is likely an artefact of the experimental approach used to extract phloem. This suggests that quantitative relationships between metabolites detected in plant pools may still be maintained despite differences in qualitative findings. Previous studies have suggested that the analysis of soluble plant pools may be used to indicate overall plant productivity and predict abiotic stresses that impact on the development of reproductive tissue [34,35,36]. The results obtained within this study suggest that the use of diagnostic tests that measure soluble pools to detect nutrient deficit may be of little benefit if they do not also reflect the impact of stress on the nutritional content of the seed. Understanding the decoupling of nutrient content between the leaf and the pod and how this process occurs over plant development would assist in developing reliable diagnostic tools for maintaining yield and nutritional quality of the resulting seed.

This study has demonstrated the impact of reduced nutrient supply on physiology, yield and nutrient content in common bean. We detected reductions in carbon assimilation with reducing amounts of nutrient supply over development, alongside similar responses in the biochemical parameters that underlie the photosynthetic pathway. Consequently, yield volume significantly declined with reductions in nutrient supply along with a reduced leaf area. Nutrient availability (amino acids and mineral nutrients) within the vegetative tissue altered over development likely a consequence of remobilisation to maintain the nutritive value of the pod. Understanding the implications of changes to nutrient supply on the nutritional composition of major staple food crops, such as common bean, is vital to preserve global nutrient security into the future.

## 4. Materials and Methods

### 4.1. Experimental Design

Common bean cultivar ‘Blue Lake’ (climbing bean) seeds were not inoculated, germinated and raised in the controlled environment growth facility at the Centre for Carbon, Water and Food, University of Sydney. Plants were transplanted and grown in 9 L pots filled with a 50:50 sand and vermiculite mix, watered daily to field capacity. Temperature was maintained at 25 °C during the light period and 15 °C during the dark period, and relative humidity at 65%. A photoperiod of 13 h (0700–2000) with an average light intensity of 400 μmol m^−2^ s^−1^ photosynthetically active radiation (PAR) provided by a high-pressure sodium light source. Carbon dioxide [CO_2_] levels in the chamber were approximately 420 ppm.

Upon transfer of plants into 9 L pots, 90 plants were randomly assigned to either a 100% (control), 50% (medium), 10% (low) nutrient treatment. From germination, plants in each treatment were provided every Monday and Friday with 1 L of nutrient solution as described by Macfarlane, et al. [37]; CaCl_2_*2H_2_O 700 µM, NaH_2_PO_4_*2H_2_O 500 µM, K_2_SO_4_1400 µM, MgSO_4_*7H_2_O 750 µM, H_3_BO_4_ 40 µM, MnSO_4_*4H_2_O 15 µM, Fe-Na-EDTA 25 µM, ZnSO_4_*7H_2_O 1 µM, CuSO_4_*5H_2_O 1 µM, CoSO_4_*7H_2_O 0.3 µM, Na_2_MoO_4_*2H_2_O 0.15 µM, NH_4_NO_3_ 6000 µM, diluted by 0.5 or 0.1 for the medium and low treatments respectively. A total 24 plants per treatment, 8 per development stage, were randomly sampled for analysis upon majority of the plant reaching the development stages–flowering (days after sowing (DAS) 42–45), mid pod fill (DAS 56–59) and pod maturity (DAS 70–73).

### 4.2. Leaf Gas Exchange Measurements

Gas exchange for each treatment and at each development stage was measured using a WALZ GFS-3000 portable infra-red gas analyser (Walz Heinz GmbH, Effeltrich, Germany). Each plant for every treatment and development stage was measured sequentially at least seven times across the light period with a different fully expanded, non-shaded leaf chosen for measurement at each time point. Light conditions were set to tracking while temperature, relative humidity, and CO_2_ mole fraction of reference air in the measuring chamber were maintained to ambient air conditions. Net CO_2_ assimilation rate (*A*, μmol m^−2^ s^−1^) and stomatal conductance to water vapour (*g_s_*, mmol m^−2^ s^−1^) were logged and the ratio of *c_i_*/*c_a_* (where *c_i_* is the sub-stomatal CO_2_ concentration in μmol mol^−1^ and *c_a_* is the ambient atmospheric CO_2_ concentration in μmol mol^−1^) was calculated.

### 4.3. Tissue Collection

At each development stage at the end of the light period on the day of the gas exchange measurements, all above and below ground plant material was harvested (only leaves and pods were used for final analysis). Phloem sap was collected according to previous methods [38,39]. In brief, phloem tissue samples were collected using a razor blade, placed into microtubes with MQ water and incubated at 4 °C for two hours after which stems were removed and the liquid frozen at −80 °C. All plant material was frozen at −80 °C awaiting further analysis.

### 4.4. Extractions of Leaf and Seed Material

Samples of leaves and pods for each development stage were oven dried at 65 °C and ground using an oscillating matrix mill. For available mineral nutrient and amino acid analysis approximately 40 mg of ground sample was then weighed into a 2 mL micro-tube and extracted in a hot water mix according to the protocol outlined in Merchant et al. [40]. An additional 20 mg of ground pod material was placed with 1 mL of 6 M hydrochloric acid in a vacuum hydrolysis tube (Thermo Scientific) and digested for 24 h at 110 °C on a heating module (Thermo Scientific, Reacti-Therm III). Products of the hot water extraction and digestion process were stored frozen at −80 °C awaiting further analysis.

### 4.5. Analysis of Carbon Isotope Abundance

Determination of carbon isotope abundance and nitrogen content (%) in ground samples of leaves for each development stage and pods at pod maturity stage, was completed according to the methods outlined in Merchant, et al. [41] using a Delta V Advantage isotope ratio mass spectrometer (IRMS) (Thermo Electron) with a Conflo IV interface (ThermoFisher Scientific, Bremen, German).

### 4.6. Analysis of Plant Material for Amino Acids and Nutrients

Determination of soluble amino acids in leaf material and total amino acids for pod material collected at pod maturity in extracted and digested samples respectively, was completed using high performance liquid chromatography coupled to a quadrupole time-of-flight mass spectrometer (HPLC-MS). HPLC separation was completed on an Agilent 1290 Infinity system (Agilent, Walbronn, Germany) using a Zorbax StableBond SB-CB18 column (150 × 2.1 mm, 3.5 μm, Agilent) including degasser, binary pump, temperature-controlled autosampler (maintained at 4 °C) and column compartment (maintained at 30 °C). The mobile phase was composed of water containing 0.1% formic acid (solution A) and methanol containing 0.1% formic acid (solution B) [42]. The flow rate was 0.3 mL min^−1^ with a gradient elution of 0 to 100% solution B, over 23 min for positive mode respectively. Amino acids were detected by a quadrupole time-of-flight mass spectrometer (Agilent 6520 QTOF accurate-mass) with a dual electrospray ionization (ESI) source. The mass spectrometer was operated with full scan in positive FT mode for amino acid analysis see, Reference [42]. ESI capillary voltage was set at 4000 V (+) ion mode and 3500 V (−) ion mode and fragmentor at 135 V. The liquid nebulizer was set to 30 psig and the N drying gas was set to a flow rate of 10 L min^−1^. Drying gas temperature was maintained at 300 °C. Internal reference ions were used to continuously maintain mass accuracy. Molecular ions ([M + H]+ for amino acids) were extracted from the full scan chromatograms and peak areas integrated using Agilent MassHunter Workstation software (Agilent Technologies, Santa Clara, CA, USA).

Determination of soluble nutrients and total nutrients in extracted and digested samples respectively, was completed using an inductively coupled plasma optical emission spectrometer (Varian Vista, Santa Clara, CA, USA). Samples were prepared with a dilution of 400 µL of supernatant in 10 mL of ultra-pure Milli-Q water. Nutrients Ca, Fe, K, Mg, P, S and Zn, were chosen for analysis. Any results lower than the detection limit of the instrument were adjusted to zero. The pod macronutrient content was calculated by the pod nutrient concentration multiplied by pod weight.

### 4.7. Statistical Analysis

Analysis of linear mixed models using the method of residual maximum likelihood (REML) was completed using GenStat 15th Edition (VSN International, Hemel, Hampstead, UK). Fishers unprotected least significant difference (LSD) test was used for post hoc testing. The pod nutrient content and gas exchange results were analysed by Tukey test at 5% probability, using the software Statistica 8.0.

## Figures and Tables

**Table 1:** Time course of average photosynthesis (A μmol m^−2^ s^−1^), stomatal conductance (g_s_ mmol m^−2^ s^−1^), the ratio of internal CO_2_ concentration (c_i_ μmol m^−1^) and atmospheric carbon concentration (c_a_ μmol m^−1^), leaf carbon isotope abundance (δ^13^C ‰) and leaf nitrogen content (%) for common bean subject to control, medium and low nutrient supply treatments over the course of development flowering, mid pod fill, pod maturity. Standard errors are shown. Statistically significant differences between treatments were detected for A, g_s_ during the pod maturity stage (denoted by multiple comparisons lettering, *p* < 0.05).

Stage	Trt	A (μmol m^−2^ s^−1^)	g_s_ (mmol m^−2^ s^−1^)	c_i/_c_a_ (μmol m^−1^)	Leaf δ^13^C (‰)	Leaf N (%)
**Flowering**	Control (100%)	8.52 ± 0.70	0.10 ± 0.01	0.60 ± 0.08	−35.2 ± 0.3	4.2 ± 0.3
	Medium (50%)	8.51 ± 0.48	0.11 ± 0.01	0.61 ± 0.10	−35.2 ± 0.4	4.0 ± 0.3
	Low (10%)	8.56 ± 0.59	0.10 ± 0.01	0.56 ± 0.11	−36.6 ± 1.1	3.3 ± 0.3
**Mid Pod Fill**	Control (100%)	5.65 ± 0.52	0.06 ± 0.01	0.53 ± 0.07	−33.3 ± 0.3	2.4 ± 0.3
	Medium (50%)	4.99 ± 0.53	0.06 ± 0.01	0.51 ± 0.05	−33.6 ± 0.6	1.9 ± 0.3
	Low (10%)	4.34 ± 0.44	0.05 ± 0.01	0.60 ± 0.06	−33.8 ± 0.5	1.4 ± 0.1
**Pod Maturity**	Control (100%)	3.18 ± 0.31a	0.04 ± 0.01ab	0.59 ± 0.08	−32.6 ± 0.5	1.3 ± 0.2
	Medium (50%)	3.06 ± 0.47a	0.06 ± 0.01a	0.73 ± 0.05	−32.4 ± 0.5	1.1 ± 0.1
	Low (10%)	1.17 ± 0.20b	0.03 ± 0.01b	0.79 ± 0.06	−31.7 ± 0.6	1.1 ± 0.1

## Data Availability

Not application.
